# Investigation on the Incidence of Syncope in Children and Adolescents Aged 2–18 Years in Changsha

**DOI:** 10.3389/fped.2021.638394

**Published:** 2021-03-22

**Authors:** Erlin Hu, Xiaoyan Liu, Qianqian Chen, Cheng Wang

**Affiliations:** ^1^Department of Pediatric, Changsha Maternal and Child Health Hospital, Hunan Normal University, Changsha, China; ^2^Department of Pediatric Cardiovasology, Children's Medical Center, The Second Xiangya Hospital, Central South University, Changsha, China; ^3^Department of Pediatric, Changsha Central Hospital, University of South China, Changsha, China

**Keywords:** syncope, incidence, children, adolescents, cross-section survey

## Abstract

**Objectives:** Syncope is a common clinical symptom, while there are less relevant literature and targeted research on childhood morbidity. This article makes a cross-section survey on the incidence of syncope in children and adolescents aged 2–18 years in Changsha.

**Materials and Methods:** There were 4,352 children and adolescents aged 2–18 years randomly selected from six primary and secondary schools and three kindergartens in Changsha from March 2018 to November 2018. There were 4,916 standardized questionnaires issued, and 4,352 (88.53%) valid questionnaires were recovered.

**Results:** (1) Incidence: 17.37% of children and adolescents aged 2–18 years who had at least more than one syncope; the incidence in the adolescence (28.85%) was higher than that in the school age (8.32%) and in the preschool age (2.71%) (*P* < 0.01). (2) Age at onset: 13.9 ± 3.1 years old, with a peak age of 16 years. (3) Gender difference: The incidence in adolescent females was higher than that in males (31.72 vs. 26.25%, *P* < 0.05). In inducements, females had higher rates than males in sweltering environment (*P* < 0.01), whereas males had higher rates than females in urination (*P* < 0.05). Dizziness, nausea, sweating, and facial pallor were higher in females than in males in presyncope (*P* < 0.05).

**Conclusions:** The incidence of syncope in children and adolescents aged 2–18 years in Changsha is 17.37%. The incidence of syncope is different between males and females in different age groups; there are gender differences in syncope inducements and presyncope.

## Introduction

Syncope is a symptom characterized by a transient and complete loss of consciousness that results in an inability to maintain muscular tension and a rapid spontaneous complete recovery. The mechanism may be cerebral hypoperfusion (1). The lifetime cumulative incidence of syncope is 35%. Syncope occurred more often in females than in males (41 vs. 28%, *P* = 0.003). A peak in the incidence of syncope occurred around the age of 15 years in both males and females (2). Although syncope is mostly benign, some patients with a syncope attack may have a high risk of sudden death (3) or lead to syncope-related bodily accidental injuries, which seriously affect children's physical and mental health and quality of life (4). In children and adolescents, many diseases can cause syncope, and 70–80% of which are autonomic neurally mediated syncope (4), such as vasovagal syncope (VVS), postural tachycardia syndrome, orthostatic hypotension, and orthostatic hypertension (5). Through guidelines for the diagnosis and treatment of syncope in children and adolescents and by standardizing the diagnostic process, most of the causes can be identified (6–8). However, there is no epidemiological investigation report of syncope in a large sample of children and adolescents. In this study, the incidence of syncope in children and adolescents aged 2–18 years in Changsha was investigated by questionnaire. The main objective was to understand the incidence of syncope among children and adolescents in the region and to explore gender and age differences.

## Methods

### Study Population

#### The Research Object

From March 2018 to November 2018, three secondary schools, three primary schools, and three kindergartens in Changsha were randomly selected from the school list of Changsha Education Bureau. There were six grade groups in each primary school and six grade groups in each secondary school. Three classes were randomly selected from each grade group, totaling 108 classes, with ~40–50 cases in each class. The kindergarten was divided into large class, middle class, and small class. A total of 21 classes were selected, with ~25–35 cases in each class. Primary and secondary school children and adolescents were 6–18 years old, and children in kindergarten were 2–6 years old. For each case included in the survey, members of the research group and the school physician conducted a simple history of illness inquiry and a routine physical examination. After the elimination of organic diseases, questionnaires were issued. Young children of preschool age and school age were assisted on site by their parents to complete the questionnaire.

#### Description of Study Subjects and Recruitment

Inclusion and exclusion criteria were in accordance with the references (9–11). The questionnaire of appropriate length was used. Easily misdiagnosed as syncope is a common clinical situation, which mainly includes some other basic diseases leading to transient loss of consciousness, including epilepsy, metabolic turbulence disorderliness, and mental factors. These diseases called “pseudo syncope” were excluded. History and physical examination are the most specific and sensitive ways to evaluate syncope (12). The diagnosis is achieved with a thorough history and physical examination alone in more than 60% of patients (13, 14).

##### Inclusion

All children and adolescents aged 2–18 years randomly selected from schools and classes were eligible for the study.

##### Exclusion

Asking for medical history and physical examination, study subjects should be excluded if any of the following conditions are met:

1. Prolonged loss of consciousness: it lasts more than 5 min.
2. Loss of representational consciousness: pseudosyncope or psychogenic syncope.
3. Posttraumatic loss of consciousness.
4. Poisoning and metabolic diseases.

##### The Syncope History Should Consist of Two Questions

1. Have you been fainted, lost consciousness, passed out, or fallen for no reason? If the answer is yes, ask further information about the onset and duration.
2. What's your past medical history?

##### Routine Physical Examination

1. Check vital signs.
2. Auscultate the heart and lungs.

##### Prevention of Selection Bias

1. Parents assisted to fill in the questionnaires for primary and kindergarten students.
2. Members of the investigation team communicated with their parents on the phone before the questionnaires were given to middle school students.

##### The Quality Control

Strict quality control was carried out during the study. The first is stratified training: first, training the members of the research group and then training the school physicians and teachers, and finally training parents and students. The second are unified inclusion and exclusion criteria, and unified medical history inquiry and physical examination. Finally, all positive syncope questionnaires were reviewed by telephone.

A total of 4,916 questionnaires were issued, of which 564 were incomplete or unqualified, and 4,352 were qualified. The recovery rate was 88.53%.

### Study Methods

#### Contents of Questionnaire

First, a preliminary questionnaire was designed according to reference (15), and then questionnaire scheme was modified according to “Syncope Evaluation in Children Investigations/Questionnaire Protocol.” The validity and reliability of the questionnaire are as follows: (1) Cronbach α = 0.724, which shows better reliability; (2) KMO number of sampling appropriateness = 0.770, which shows good validity. Questionnaire contents include (1) general data: gender, age, body mass, and height; (2) syncope frequency, which was identified as the total number of syncope episodes before completing the syncope questionnaire; (3) triggers: prolonged standing, sweltering environment (an environment with high temperature at high relative humidity), exercise, defecation, and urination; (4) presyncope: dizziness, headache and facial pallor; (5) syncope position: standing position, prone position, and sitting position; (6) duration of syncope: several seconds, <5, 5–10, and >10 min; (7) accompanying symptoms: twitching of limbs, urinary and fecal incontinence, body injuries; (8) postsyncope status: weakness, dizziness, and headache; (9) medical history and family history: past diseases, type of disease, and history of syncope in the immediate family; (10) duration of and age at the first syncope onset.

### Data Collection

Each questionnaire was provided with information on how to fill in the form and general knowledge of syncope, and a consulting phone number was available on the form. A training course was organized for investigators before the survey to unify investigation methods. In every school, it was arranged that a research team member checked each questionnaire for ensuring the quality. After that, team members inspected 5% of the questionnaires, and 45.9% of the questionnaires were further verified by telephone with parents. Finally, all the data were entered into the computer by the especially assigned person after the review and logical analysis.

### Statistics Process

All data were statistically analyzed with SPSS 17.0 software (IBM Corp, Armonk, NY). The data were presented as mean ± SD, and the *t*-test was used for comparison between groups. Categorical data were represented by percentile (%). χ^2^ Test was applied for comparison among groups, and *P* < 0.05 indicated statistical significance.

## Results

### General Information

Among the 4,352 cases, 52.39% (2,280/4,352) were male, and 47.61% (2,072/4,352) were female. The ages ranged from 2 to 18 years (mean, 10.9 ± 4.3 years old). Among them, 627 cases (14.41%) were at the age of 2–5 years, 1,635 (37.57%) were 6–11 years old, and 2,090 (48.02%) were 12–18 years old.

### Incidence

#### Incidence Rate

**Figure 1:**
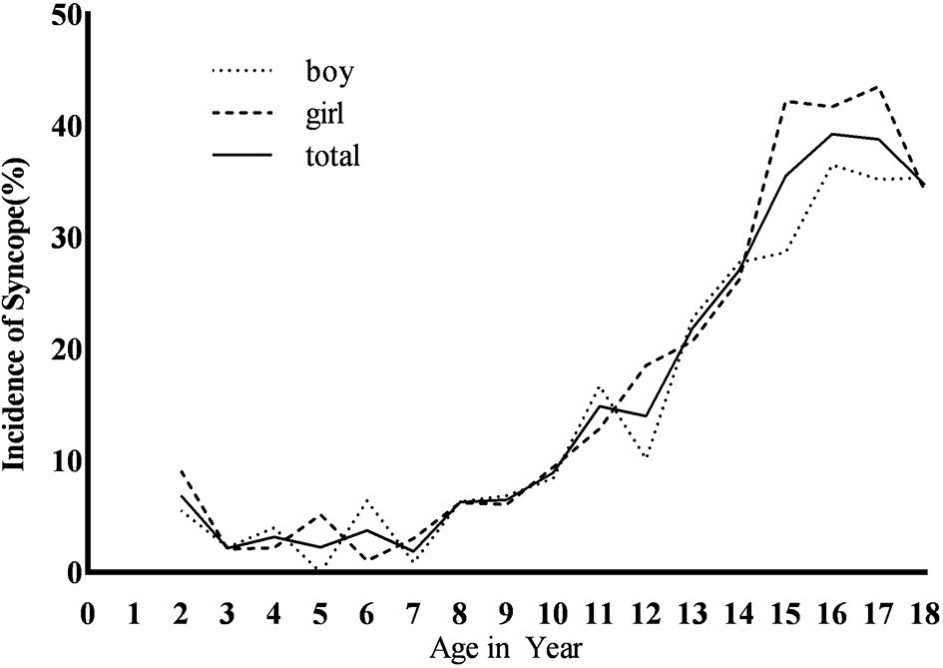
The incidence of syncope at age span of 2–18 years in Changsha.

This study was a status survey. Whether the subjects had syncope before filling in the questionnaire was investigated. When calculating the incidence of syncope, only one syncope is counted, regardless of the number of episodes of syncope. Among the 4,352 subjects, 756 cases (17.37%) had at least one experience of syncope (Figure 1), with 373 males (49.34%) and 383 females (50.66%). There was no statistical significance between males and females (χ^2^ = 3.41, *P* > 0.05).

#### Age Difference

The average age at onset was 13.9 ± 3.1 years, and the incidence increased with age from 7 years old, with the peak age of 16 years. There was statistical significance in the incidence of syncope among the three age groups (χ^2^ = 379.15, *P* < 0.01). The adolescence was of higher incidence than the school age [28.85% (603/2,090) vs. 8.32% (136/1,635), χ^2^ = 243.21, *P* < 0.01] and the preschool age [28.85% (603/2,090) vs. 2.71% (17/627), χ^2^ = 187.13, *P* < 0.01]. The school age was of higher syncope incidence than the preschool age [8.32% (136/1,635) vs. 2.71% (17/627), χ^2^ = 22.59, *P* < 0.01] (Table 1 and Figure 1).

**Table 1:** Frequency table about syncope age of children and adolescents in Changsha.

**Age (year)**	**2**	**3**	**4**	**5**	**6**	**7**	**8**	**9**	**10**	**11**	**12**	**13**	**14**	**15**	**16**	**17**	**18**	**Total**
Number of cases	29	276	189	133	186	210	300	214	269	456	399	370	266	304	331	250	170	4,352
Number of syncope	2	6	6	3	7	4	19	14	24	68	56	81	72	108	130	97	59	756
Morbidity (%)	6.89	2.17	3.17	2.26	3.76	1.90	6.33	6.54	8.92	14.91	14.04	21.89	27.07	35.53	39.27	38.80	34.71	17.37

#### Gender Difference

In the adolescence, the incidence of females was higher than that of males [31.69% (315/994 cases) vs. (26.28%, 288/1,096 cases), χ^2^ = 7.44, *P* < 0.05]. There was no statistical significance in syncope incidence between males and females at school age [8.98% (77/859 cases) vs. 7.58% (59/776 cases), χ^2^ = 0.99, *P* > 0.05] and preschool age [2.46% (8/325 cases) vs. 3.00 (9/302 cases), χ^2^ = 0.16, *P* > 0.05], and no statistical significance in the incidence of males and females in the three age groups (χ^2^ = 3.41, *P* > 0.05; Figure 1).

### Syncope Triggers

Common triggers for syncope are prolonged standing, sweltering environment, and exercise. In terms of gender comparison of syncope triggers, females were of higher incidence than males in sweltering environment (*P* < 0.05), whereas males had higher rates than females in urination (*P* < 0.05). The results are shown in Table 2.

**Table 2:** Gender comparison of the syncope triggers about children and adolescents in Changsha.

**Inducement**	**Male *n* (%)**	**Female *n* (%)**	**Total**	**χ^2^**	***P***
Prolonged standing	179 (47.99)	208 (54.31)	387	3.02	0.08
Sweltering environment	84 (22.52)	124 (32.38)	208	9.20	0.02
Exercise	56 (15.01)	66 (17.23)	122	0.69	0.41
Severe pain	24 (6.43)	20 (5.22)	44	0.51	0.48
Mental stimulation	19 (5.09)	23 (6.00)	42	0.30	0.58
Catch sight of blood	17 (4.56)	19 (4.96)	36	0.07	0.79
Hot bath	13 (3.49)	14 (3.66)	27	0.02	0.90
Defecation	14 (3.75)	8 (2.09)	22	1.85	0.17
Urination	10 (2.68)	2 (0.52)	12	5.64	0.02

### Presyncope

Dizziness and blurred vision appeared more frequently in symptoms of presyncope. And symptoms of dizziness, facial pallor, sweating, and nausea were higher in females than in males for presyncope (*P* < 0.05; Table 3).

**Table 3:** Gender comparison of the presyncope about children and adolescents in Changsha.

**Presyncope**	**Male *n* (%)**	**Female *n* (%)**	**Total**	**χ^2^**	***P***
Dizziness	224 (60.05)	261 (68.15)	485	5.38	0.02
Blurred vision	107 (28.69)	134 (34.99)	241	3.45	0.06
Facial pallor	37 (9.92)	80 (20.89)	117	17.38	<0.01
Profuse sweat	36 (9.65)	69 (18.02)	105	11.05	<0.001
Headache	50 (13.40)	46 (12.01)	96	0.33	0.56
Hearing loss	50 (13.40)	43 (11.23)	93	0.83	0.36
Nausea	27 (7.24)	61 (15.93)	88	13.87	0.01
Vomiting	23 (6.17)	31 (8.09)	54	1.06	0.30
Slow response	25 (6.70)	25 (6.53)	50	0.01	0.92
Cyanosis of lips	11 (2.95)	14 (3.66)	25	0.29	0.59

### Other

#### Frequency of Syncope

The frequency of syncope was identified as the total number of syncope; 55.56% of cases (420/756) had only one episode of syncope; 44.31% of cases (335/756) had two or more episodes of syncope.

#### Position and Duration of Syncope

The main syncope positions were standing (75.66%, 572/756) and sitting (18.65%, 141/756). The duration of syncope was 79.63% (602/756) at <5 min, and 51.85% (392/756) were within a few seconds.

#### Symptoms After Syncope

The main symptoms after syncope were dizziness (47.22%, 357/756), weakness (35.05%, 265/756), and headache (10.19%, 77/756). Some children and adolescents developed more than two of these symptoms after syncope.

#### Age at the First Syncope and Family History of Syncope Onset

Average age at the first syncope onset was 12.6 ± 3.8 years; 5.69% (43/756) of children and adolescents with syncope experience had history of syncope in their first-degree relatives.

## Discussion

Syncope is a common emergency in children and adolescents, accounting for 1–2% of all pediatric emergencies (16). Syncope incidence in children and adolescents is increased year by year. From 1950 to 1954, it was 71.9 per 100,000, and from the late 1980s to the early 1990s, it rose to 125.8 per 100,000 (17). Ruwald et al. (18) reported that in Denmark 127,508 patients with the diagnosis of syncope were involved in a 12-year observational study; the incidence of syncope increased from 13.8‰ in 1997 to 19.4‰ in 2009. Bo et al. (19) reported that the incidence of syncope in the pediatric population of Parma during a 2-year period (2005–2006) resulted in 86.5 per 100,000 per year. This article, through a questionnaire survey of 4,352 children aged 2–18 years, showed that the incidence of syncope was 17.37%. The incidence was defined as the proportion of children aged 2–18 years who were finally diagnosed with syncope in the total population during the trial phase. This was the baseline incidence of syncope during the survey period. The incidence rate was significantly higher in the 12–18-year age group, which was roughly the same age and incidence of reflex syncope as reported by Serletis et al. (20). There are age differences in incidence of syncope. In this study, the incidence increased with age from 7 years old. The incidence was 28.87% in the adolescence, 8.02% in the school age, and 2.69% in the preschool age. The average age at onset was 13.9 ± 3.1 years, and the peak age was 16 years old. It is basically consistent with the average age (14.7 years old) and peak age at onset (13–18 years old) of syncope children reported by Anderson et al. (21).

As for the gender differences of syncope, Ganzeboom et al. (22) reported the cumulative incidence of syncope was almost twice as high in females as in males (47 vs. 24%). Romme et al. (23) reported that most of the triggers and prodrome were more common in younger patients and females. Ruwald et al. (18) found that the age distribution of the patients showed three peaks around 20, 60, and 80 years of age. The first peak was represented primarily by females at ~20 years of age; the third peak occurred earlier in males than females. This study showed that in terms of gender, only females aged 12–18 years were higher in incidence than males, and there was no statistical significance between males and females in the overall investigated population and groups aged 6–11 and 2–5 years, suggesting that there be no gender difference in syncope of younger age groups.

Sex and age differences of syncope may be related to hormone levels and sympathetic nervous system development. Human and animal data indicate significant differences existing between males and females in basal function of the autonomic nervous system (24). Females have higher parasympathetic, cardiac autonomic activity. Gonadal hormones play a role in sexual dimorphism of autonomic control. Both estrogen and testosterone are known to modulate the synthesis of catecholamines, the primary transmitter of the sympathetic nervous system. Furthermore, there is evidence that autonomic indices vary significantly across the menstrual cycle. Parasympathetic activity is more prominent in the luteal phase, suggesting a key role for estrogen in the regulation of parasympathetic activity (25). Anderson et al. (21) reported syncope patients of emergency departments in all medical institutions in the United States. Among children and adolescents with syncope, 20% were aged 7–12 years, 80% were aged 13–18 years, and the majority of syncope patients were female (70.1%). It is basically consistent with Shim et al. (26). For gonadal hormones, which play a role in sexual dimorphism of autonomic control, there is no sex difference between preadolescence and postmenopausal syncope (27).

Most incidence of syncope was triggered; as a result of these inducements including prolonged standing, sweltering environment, and exercise, the male had a reduced returned blood volume to the heart, resulting in syncope. For syncope triggered by sweltering environment, rates in females were obviously higher than those in males, which may be related to the relatively sex difference in autonomic nervous function, whereas urination-induced syncope was higher in males than in females, which was consistent with the results reported by Komatsu et al. (28) and Bae et al. (29). It may be related to the different urination postures between males and females. Males take a standing posture to urinate. When standing still, the lower-extremity muscles do not perform rhythmic relaxation and contraction, but maintain a state of tension and contraction. Then the veins will continue to be compressed, and the venous return will decrease, which reduces the return blood volume and is prone to short-term cerebral blood supply. Females urinate in a squatting posture, and the veins of the lower extremities are compressed, which promotes blood return; in the squatting position, the abdominal wall is passively compressed, and the pressure in the abdominal cavity increases, which also promotes the increase of the return blood volume. However, there was no gender difference in defecation syncope, which may be related to age, as according to Bae et al. (29), defecation syncope is more common in elderly women, whereas the subjects of this study were children between 12 and 18 years.

Syncope episodes are with not only triggers, but also premonitory signs. Noizet-Yverneau et al. (30) prospectively studied the data of 159 pediatric emergency patients (mean age, 11 ± 4 years) for more than 1 year and found that the incidence of presyncope was 52%. Paris et al. (31) used a standardized clinical assessment and management plan for outpatients aged 7–21 years who were first diagnosed with syncope and found that more than 80% of the patients had dizziness or lightheadedness and experienced a prodrome. In this study, presyncope was characterized by dizziness, blurred vision, and pale complexion. At the same time, there were differences in the presyncope between males and females, and the symptoms of presyncope were more in females than in males, which may be related to higher parasympathetic and cardiac autonomic activity in females.

Loss of consciousness is usually due to seizures, syncope, drop attacks, metabolic turbulence disorderliness, and mental factors. Some babies have breath-hold seizures. Drop attacks involve sudden falls without consciousness or warning and with immediate recovery. The typical signs of epileptic seizures include tongue lacerations, limb jerking, and postictal confusion, but not common signs of syncope, such as prodromal diaphoresis, palpitations, or provocation by prolonged sitting or standing (32). Metabolic syncope due to hypoglycemia is diagnosed when the loss of consciousness is preceded by tremors, confusion, salivation, hunger, and a hyperadrenergic state, and glycemia is <40 mg/dL. VVS has three distinct phases: a prodrome, loss of consciousness, and a postsyncopal phase. The assessment of syncope in children and adolescents is more complex, but history and physical examination are the most specific and sensitive ways to evaluate syncope. In this study, a detailed history and physical examination were conducted; admission and exclusion criteria were strictly controlled to obtain the baseline incidence of syncope and age and sex differences in children aged 2–18 years in Changsha city. Therefore, the evaluation of syncope focused on a detailed history and physical examination (12). The history should focus on circumstances immediately before the attack, its onset, the attack, the end of the attack, and the patient's background. The physical examination should focus on vital signs, including measures of supine and standing blood pressure and the cardiovascular and neurologic systems.

In conclusion, this study revealed the epidemiological characteristics of syncope in children and adolescents aged 2–18 years in Changsha, and there were differences in the incidence and gender among different age groups, as well as gender differences in common syncope inducements and presyncope, which provided some objective basis for effective intervention measures.

## Study Limitations

The limitation one is that the questionnaire does not contain the following contents:

Had the child taken anything to eat or drink?

Had the child taken any medication?

Was the syncope during exercise? (It is important to define whether the syncope is actually during the exercise, which may suggest a cardiac cause).

The lack of these three items in the questionnaire may affect etiological analysis, such as poisoning and organic heart disease.

Next limitation is rate of return. Although the research method is random sampling, there may be random sampling error. The rate of the questionnaire return visits was 45.9%, which may also affect the accuracy of the results. Despite the above possible error probability, the results of this study reflect a trend in the incidence of unexplained syncope in children and adolescents aged 2–18 years.

## Data Availability Statement

The data analyzed in this study is subject to the following licenses/restrictions: The datasets generated for this study are available on request to the corresponding author. Requests to access these datasets should be directed to wangcheng2nd@csu.edu.cn.

## Ethics Statement

The studies involving human participants were reviewed and approved by the study was approved by the Ethics Committee of The Second Xiangya Hospital, Central South University. Written informed consent to participate in this study was provided by the participants' legal guardian/next of kin. Written informed consent was obtained from the minor(s)' legal guardian/next of kin for the publication of any potentially identifiable images or data included in this article.

## Author Contributions

EH and CW conceptualized and designed the study, performed the data analysis, interpreted the study findings, drafted the initial manuscript, and revised the manuscript. QC interpreted the study findings. XL reviewed and revised the manuscript. All authors approved the final manuscript as submitted and agree to be accountable for all aspects of work.

## Conflict of Interest

The authors declare that the research was conducted in the absence of any commercial or financial relationships that could be construed as a potential conflict of interest.
